# Performance of Myocardial Perfusion Imaging Using Multi-focus Fan Beam Collimator with Resolution Recovery Reconstruction in a Comparison with Conventional SPECT

**Published:** 2014

**Authors:** Norikazu Matsutomo, Akio Nagaki, Masayuki Sasaki

**Affiliations:** 1 Department of Radiology, Kurashiki Central Hospital, Kurashiki, Japan; 2 Department of Health Sciences, Graduate School of Medical Sciences, Kyushu University, Fukuoka, Japan

**Keywords:** Multi-focus fan beam collimator, Myocardial perfusion imaging, Comparable images, Resolution recovery reconstru-ction

## Abstract

**Objective(s):**

IQ-SPECT is an advanced high-speed SPECT modality for myocardial perfusion imaging (MPI), which uses a multi-focus fan beam collimator with resolution recovery reconstruction. The aim of this study was to compare IQ-SPECT with conventional SPECT in terms of performance, based on standard clinical protocols. In addition, we examined the concordance between conventional and IQ_SPECT in patients with coronary artery disease (CAD).

**Methods:**

Fifty-three patients, undergoing rest-gated MPI for the evaluation of known or suspected CAD, were enrolled in this study. In each patient, conventional SPECT (^99m^Tc-tetrofosmin, 9.6 min and ^201^Tl, 12.9 min) was performed, immediately followed by IQ-SPECT, using a short acquisition time (4.3 min for ^99m^Tc-tetrofosmin and 6.2 min for ^201^Tl). A quantitative analysis was performed on an MPI polar map, using a 20-segment model of the left ventricle. An automated analysis by gated SPECT was carried out to determine the left ventricular volume and function including end-diastolic volume (EDV), end-systolic volume (ESV), and left ventricular ejection fraction (LVEF). The degree of concordance between conventional SPECT and IQ-SPECT images was evaluated according to linear regression and Bland-Altman analyses.

**Results:**

The segmental percent uptake exhibited a significant correlation between IQ-SPECT and conventional SPECT (*P*<0.05). The mean differences in ^99m^Tc-tetrofosmin studies were 1.1±6.6% (apex), 2.8±5.7% (anterior wall), 2.9±6.2% (septal wall), 4.9±6.7% (lateral wall), and 1.8±5.6% (inferior wall). Meanwhile, regarding the ^201^Tl-SPECT studies, these values were 1.6±6.9%, 2.0±6.6%, 2.1±5.9%, 3.3±7.2%, and 2.4±5.8%, respectively. Although the mean LVEF in IQ-SPECT tended to be higher than that observed in conventional SPECT (conventional SPECT=64.8±11.8% and IQ-SPECT=68.3±12.1% for ^99m^Tc-tetrofosmin; conventional SPECT= 56.0±11.7% and IQ-SPECT=61.5±12.2% for ^201^Tl), quantitative parameters were not significantly different between IQ-SPECT and conventional SPECT.

**Conclusion:**

According to the ^99m^Tc-tetrofosmin and ^201^Tl protocols, IQ-SPECT images were comparable to and in agreement with conventional SPECT images. Our results suggest that IQ-SPECT is a useful technology for MPI SPECT, and can lead to an increase in scan efficiency and patient comfort.

## Introduction

Myocardial perfusion imaging (MPI) is currently the most widely performed imaging modality for the diagnosis of ischemic heart disease (1-5). However, MPI requires a relatively long acquisition time for securing sufficient image quality for clinical purposes. In addition, long acquisition time results in image artifacts (e.g., motion artifacts), particularly in severely ill or older patients.

Advances in MPI technology have provided an opportunity for improved image quality, patient comfort, and throughput. In this regard, Asao et al. (6) reported that the interpolated projection data estimation processing (IPDE) method improves the image quality of MPI single-photon emission computerized tomo-graphy (SPECT), given the short acquisition time.

Borges-Neto et al. (7), Sun et al. (8), and Venero et al. (9) showed that a reconstruction algorithm, employing collimator distance-dependent resolution recovery, is expected to have the potential to decrease the acquisition time and improve diagnostic accuracy. However, these results were simply due to the effects of image processing and/or application of resolution recovery method; in fact, the findings were not associated with acquisition count.

The most basic factor required to obtain good image quality, using SPECT, is the acquired count since SPECT image quality is determined by signal-to-noise ratio. Therefore, development of a new SPECT system for obtaining a high acquisition count is required to acquire high quality images.

IQ-SPECT (Siemens AG, Erlangen, Germany) is a recently developed and advanced high-sensitivity SPECT technology that is expected to improve the efficiency of MPI. IQ-SPECT consists of three advanced features including a multi-focus fan beam collimator, cardio-centric acquisition and three-dimensional reconstruc-tion with collimator geometry (10).

The multi-focus fan beam collimator is designed in a way that the center of the field of view magnifies the heart. In addition, the collimator varies similar to a cone beam in the center, parallel to the edges; this structure contributes to the ability of collimator to increase the count. The cardio-centric imaging device rotates around a center of rotation, positioned in the heart (in a highly sensitive area of the collimator). The IQ reconstruction algorithm takes into account the unique position and shape of the collimator holes as well as the orientation of the detectors. Therefore, IQ-SPECT images are routinely reconstructed with resolution recovery and geometric correction of multi-focus collimator.

These unique features have the potential to increase the photon-sensitivity with no associated loss of resolution. Our group has previously reported that IQ-SPECT significantly improved image resolution and quality, compared to conventional SPECT in various physical phantom studies (11). However, the image accuracy and performance of IQ-SPECT has not been clinically examined yet.

In the present study, we compared IQ-SPECT with conventional SPECT in terms of performance, based on standard clinical protocols. In addition, we examined the concordance between IQ-SPECT images and conventional SPECT images in patients with coronary artery disease (CAD).

## Methods

### Patient population

Fifty-three patients (38 males and 15 females) with the mean age of 70.2 ± 11.0 years (within the range of 40-90 years), undergoing rest-gated MPI for the evaluation of known or suspected CAD, were enrolled in this study. The included patients were those with a previous history of percutaneous coronary intervention or myocardial infarction. The patients’ characteristics are shown in Table 1.

**Table 1:** Characteristics of the patient population

	^99m^TC	^201^Tl
Age(y)		
Mean±SD	71.3 ± 12.1	69.7 ± 10.0
Sex		
Male	18	20
Female	7	8
Final diagnoses		
AP	13	14
OMI	6	10
unspecified IHD	6	4
History of percutaneous coronary intervention		
	12	21

AP: angina pectoris, OMI: old myocardial Infarction IHD: ischemic heart disease

**Figure 1:**
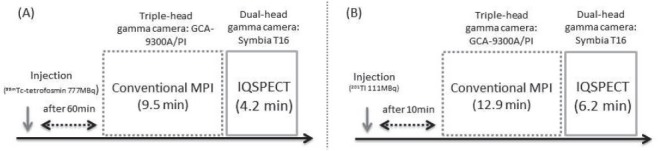
Schematic drawing of MPI SPECT, using ^99m^Tc-tetrofosmin (A) and ^201^Tl (B). All images were initially acquired by conventional SPECT, immediately followed by IQ-SPECT

All patients were initially examined using conventional SPECT, followed immediately by IQ-SPECT (Figure 1). ^99m^Tc-tetrofosmin MPI SPECT was performed on 25 patients, and ^201^Tl SPECT was carried out on 28 patients.

For the ^99m^Tc-tetrofosmin studies, 777 MBq of ^99m^Tc-tetrofosmin was intravenously injected at rest, and SPECT was initiated approximately 60 min after the injection. For the ^201^Tl studies, 111 MBq of ^201^Tl was administered intravenously, and the imaging began 15 min after the injection.

The study protocol was approved by the local ethics committee, and informed consents were obtained from all the participants.

### Conventional SPECT acquisition and reconstruction

Conventional SPECT images were acquired using a triple-head gamma camera (GCA-9300A/PI, Toshiba Medical Systems, Tokyo, Japan), equipped with a low-energy high-resolution, parallel hole collimator. The projection data were obtained using a 64×64 matrix through a 360° rotation at a step size of 6° with an acquisition time of 25 and 35 sec per projection (for ^99m^Tc-tetrofosmin and ^201^Tl, respectively), resulting in a total acquisition time of 9.6 min for ^99m^Tc and 12.9 min for ^201^Tl (including the rotation time between the successive angles).

For ^99m^Tc-tetrofosmin, a 20% energy window was centered on 140 keV photopeak and for ^201^Tl imaging, a 20% energy window, centered on 70 keV peak was used. All studies were electrocardiograph (ECG)-gated at eight frames per R-R interval. The SPECT images were reconstructed on a commercially available workstation, using filtered-back projection with a ramp filter. Also, the Butterworth filter (order=8, cut-off frequency of 0.45 cycle/cm) was used as a pre-processing filter. Short-axis images as well as vertical and horizontal long-axis images were acquired without attenuation or scatter correction.

### IQ-SPECT acquisition and reconstruction

IQ-SPECT was performed using a dual-head gamma camera (Symbia T16, Siemens AG, Erlangen, Germany) with IQ-SPECT modifications and a multi-focus fan beam collimator (Smart Zoom).

The projection data sets were acquired over 208° cardio-centric orbits with 17 views per detector for 9 sec (^99m^Tc-tetrofosmin) or 14 sec per projection (^201^Tl), resulting in a total acquisition time of 4.3 min for ^99m^Tc and 6.2 min for ^201^Tl (including the rotation time between the successive angles) on a 128×128 matrix.

For ^99m^Tc-tetrofosmin, a 15% energy window was centered on 140 keV photopeak and for ^201^Tl imaging, a 15% energy window, centered on 70 keV peak was used. All studies were ECG-gated at eight frames per R-R interval. The SPECT images were reconstructed using ordered subset conjugates-gradient minimization with 30 iterations and one subset. The reconstructed images were post-smoothed using a 3D Gaussian spatial filter (FWHM= 13 mm); neither scatter nor attenuation correction was performed.

### Quantitative analysis

A quantitative analysis was performed on an MPI polar map, using a reference to 20-segment model of the left ventricle, recommended by American Society of Nuclear Cardiology. The polar map was generated using a commercially available software program (Cedars QGS/QPS, Cedars-Sinai Medical Center) (12). The myocardial uptake was normalized to the peak activity, and the relative percent uptake of each segment was determined.

We also grouped the 20 segments into five regions: apex (segments 19 and 20), anterior (segments 1, 2, 7, 8, 13, and 14), septal (segments 3, 9, and 15), lateral (segments 5, 6, 11, 12, 17, and 18) and inferior (segments 4, 10, and 16) regions. In addition, an automated analysis was performed on gated SPECT images to determine the left ventricular (LV) volume and function including the end-diastolic volume (EDV), end-systolic volume (ESV), and left ventricular ejection fraction (LVEF).

### Statistical analysis

The degree of concordance between the conventional SPECT and IQ-SPECT images was evaluated using linear regression and Bland-Altman analyses. All IQ-SPECT parameters (percent uptake, EDV, ESV, and LVEF) were compared with those of conventional SPECT, using a paired t-test. P-value less than 0.05 was considered statistically significant, and confidence interval was determined as 95%.

## Results

### Segmental percent uptake

The interclass correlation coefficients for segmental uptake between conventional SPECT and IQ-SPECT are shown in Table 2. The interclass correlation coefficients for the ^99m^Tc-tetrofosmin segmental uptake were 0.891 (apex), 0.912 (anterior wall), 0.906 (septal wall), 0.841 (lateral wall), and 0.863 (inferior wall). Meanwhile, these values for ^201^Tl segmental uptake were 0.879, 0.885, 0.922, 0.881, and 0.909, respectively (Table 2).

**Table 2:** Comparison of the percent segmental uptake between IQSPECT and conventional SPECT

	^99m^Tc-tetrofosmin study			^201^Tl study		
	% uptake		Correlation coefficient	% uptake		Correlation coefficient
	Conventional SPECT	IQSPECT		Conventional SPECT	IQSPECT	
Apex	79.9±11.2	78.8±14.1	0.891	76.3±12.2	74.7±14.4	0.879
Anterior	73.6±13.6	70.8±13.6	0.912	73.4±13.5	71.3±14.0	0.885
Septal	68.4±12.2	65.5±14.6	0.906	66.7±14.6	64.6±15.2	0.922
Lateral	81.3±11.7	76.4±12.0	0.841	76.7±14.2	73.4±15.2	0.881
Inferior	65.5±9.8	67.7±11.1	0.863	64.0±13.5	61.6±13.6	0.909

Mean±SD

The percent uptake values showed no significant differences between conventional SPECT and IQ-SPECT images.

**Figure 2:**
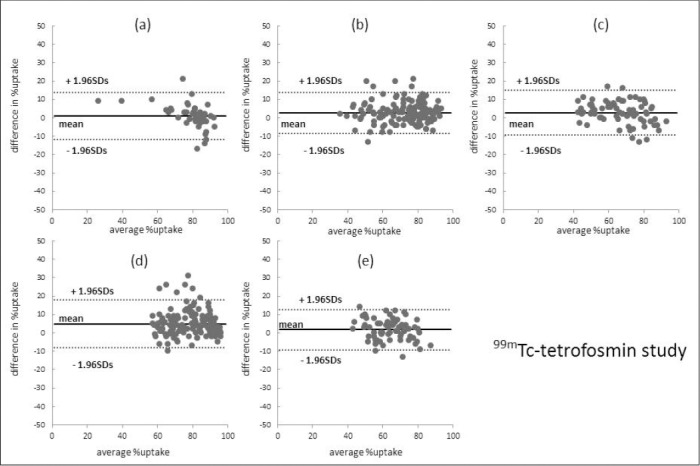
Bland-Altman analysis of the segmental percent uptake on IQ-SPECT and conventional SPECT in ^99m^Tc-tetrofosmin studies. a) apex, b) anterior, c) septal, d) lateral, and e) inferior. The mean difference in the lateral wall between IQ-SPECT and conventional SPECT slightly increased

**Figure 3:**
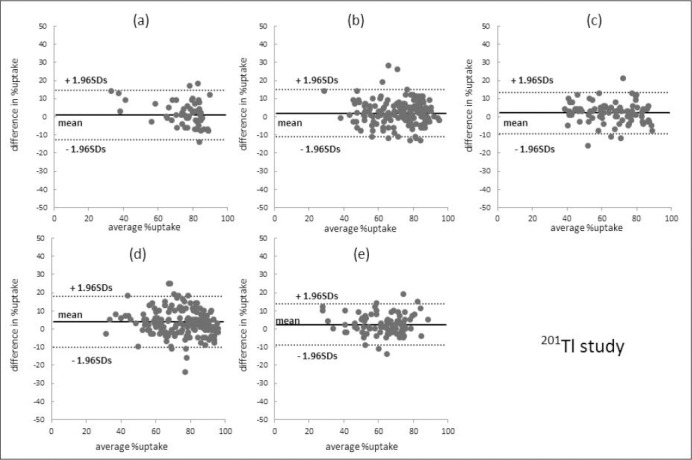
Bland-Altman analysis of the segmental percent uptake on IQ-SPECT and conventional SPECT in ^201^Tl studies. a) apex, b) anterior, c) septal, d) lateral, and e) inferior. The mean difference in the lateral wall between IQ-SPECT and conventional SPECT slightly increased

Figures 2 and 3 present the results of Bland-Altman analysis of segmental percent uptake in IQ-SPECT and conventional SPECT. The mean differences in the ^99m^Tc-tetrofosmin studies were 1.1 ± 6.6% (apex), 2.8 ± 5.7% (anterior wall), 2.9 ± 6.2% (septal wall), 4.9 ± 6.7% (lateral wall), and 1.8 ± 5.6% (inferior wall). For the ^201^Tl studies, these values were 1.6 ± 6.9%, 2.0 ± 6.6%, 2.1 ± 5.9%, 3.3 ± 7.2%, and 2.4 ± 5.8%, respectively.

**Figure 4:**
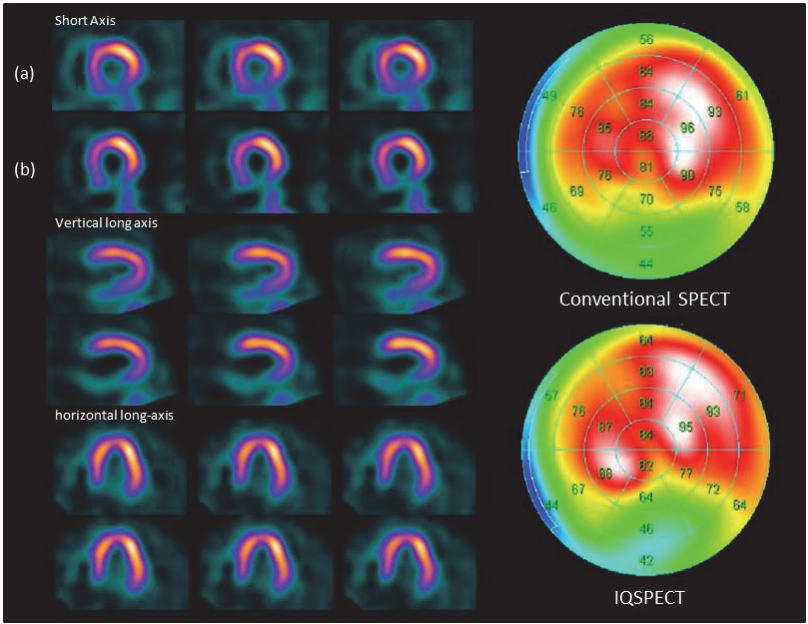
^99m^Tc-tetrofosmin MPI SPECT of a 74-year-old male with an old myocardial infarction in the inferobasal wall. Both IQ-SPECT and conventional SPECT show perfusion defect in the inferobasal wall; (a) conventional SPECT, (b) IQ-SPECT

There were no changes in the distribution of differences in the mean values. However, the mean difference between IQ-SPECT and conventional SPECT slightly increased in the lateral wall. Representative cases of MPI, evaluated with both IQ-SPECT and conventional SPECT, are presented in Figure 4. The perfusion defects were similar on both IQ-SPECT and conventional SPECT images.

### LV volume and ejection fraction

**Figure 5:**
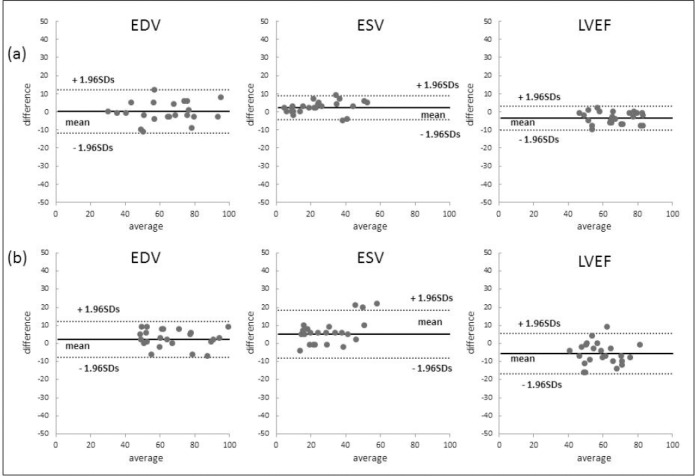
Bland-Altman analysis of the LV volume and ejection fraction on IQ-SPECT and conventional SPECT; (a) ^99m^Tc-tetrofosmin study, (b) ^201^Tl study. The mean ESV on IQ-SPECT was lower than that observed on conventional SPECT. In addition, there was a trend toward a higher mean LVEF on IQ-SPECT, compared to conventional SPECT

The correlations between EDV, ESV, and LVEF values on IQ-SPECT and conventional SPECT are shown in Table 3 and Figure 5. The correlation coefficients for the ^99m^Tc-tetrofosmin studies were 0.961 (EDV), 0.975 (ESV), and 0.960 (LVEF). For the ^201^Tl SPECT, these values were 0.966, 0911, and 0.888, respectively (Table 3).

**Table 3:** Comparison of the EDV, ESV, and LVEF values between IQSPECT and conventional SPECT

	^99m^Tc-tetrofosmin study			^201^Tl study		
	Conventional SPECT	IQSPECT	Correlation coefficient	Conventional SPECT	IQSPECT	Correlation coefficient
EDV [ml]	66.6±21.6	66.4±20.0	0.961	71.4±19.0	69.1±19.1	0.966
ESV [ml]	25.5±15.0	23.0±14.1	0.975	32.9±16.2	27.5±14.2	0.911
LVEF [%]	64.8±11.8	68.3±12.1	0.960	56.0±11.7	61.5±12.2	0.888

Mean±SD

These parameters were not significantly different between IQ-SPECT and conventional SPECT. Although the mean ESV of IQ-SPECT tended to be slightly lower than that observed in conventional SPECT, none of the parameters were significantly different between IQ-SPECT and conventional SPECT (^99m^Tc-tetrofosmin, *p*=0.614; ^201^Tl, *p*=0.174). The mean differences of ESV in the ^99m^Tc-tetrofosmin SPECT were 0.2 ± 6.0 ml (EDV) and 2.4 ± 3.3 ml. For the ^201^Tl studies, these values were 2.3 ± 5.0 ml and 5.3 ± 6.7 ml, respectively (Figure 5).

The LVEF values of IQ-SPECT and conventional SPECT showed significant correlations with respect to ^99m^Tc-tetrofosmin and ^201^Tl protocols. Although the mean LVEF of IQ-SPECT tended to be higher than that observed in conventional SPECT, the difference was not significant (^99m^Tc-tetrofosmin, *P*=0.269; ^201^Tl, *p*=0.069). The mean differences measured in the Bland-Altman analysis were -3.5 ± 3.4% (^99m^Tc-tetrofosmin) and -5.5 ± 5.7% (^201^Tl) (Figure 5).

## Discussion

In this study, we evaluated the concordance of MPI images between on IQ-SPECT versus conventional SPECT using ^99m^Tc-tetrofosmin and ^201^Tl protocols. The segmental percent uptake exhibited a significant correlation (*P*<0.05) between IQ-SPECT and conventional SPECT; the mean difference was approximately zero in the Bland-Altman analysis. In addition, quantitative parameters (EDV, ESV, and LVEF) were not significantly different between IQ-SPECT and conventional SPECT. Our results indicate that image accuracy and reproducibility of IQ-SPECT with a short acquisition time are similar to those of conventional MPI SPECT.

Regarding the segmental percent uptake, the lateral wall uptakes tended to be lower in IQ-SPECT compared to conventional SPECT. IQ-SPECT employs cardio-centric acquisition, and the acquisition angle is limited to a 208° arc. Bax et al. (13) and Go et al. (14) observed that enhanced image contrast with narrow acquisition results in geometric image distortion. In addition, Liu et al. (15) reported that a narrow acquisition orbit (e.g., 180° acquisition) yields a higher image contrast, compared to 360° acquisition in reconstructed images; in particular, the target objects are off-set on the field of view.

Our results demonstrated a similar tendency to the previously described effects due to the use of acquisition orbits. However, the differences between IQ-SPECT and conventional SPECT were slightly less significant in this study, and there were no significant differences in the level of segmental uptake. We conclude that the differential segmental uptake caused by the acquisition orbits does not affect image uniformity or homogeneity.

We also found that the LV volume (EDV and ESV) of IQ-SPECT was very similar to that observed in conventional SPECT. However, the mean LVEF of IQ-SPECT was slightly higher than that of conventional SPECT images in Bland-Altman plot. This finding demonstrates the higher image resolution of IQ-SPECT, compared to conventional SPECT.

Similarly, Onishi et al. (11) reported that the use of IQ-SPECT is more effective than conventional SPECT in terms of improving image resolution. The IQ-SPECT reconstruction method employs an iterative reconstruction algorithm with resolution recovery and a collimator geometric response function. Therefore, the application of resolution recovery algorithm reduces the partial volume effect, which seems to result in an increase in image contrast (16, 17). Despite the improvement of image contrast observed in IQ-SPECT, the increase in LVEF is insignificant and does not influence the diagnosis.

We compared the efficacy of IQ-SPECT, using a short acquisition time, with that of conventional SPECT, employing a standard acquisition time. Our results showed that accuracy and reproducibility of IQ-SPECT with a short acquisition time is similar to that observed in conventional SPECT.

In general, the SPECT protocol is determined according to the trade-off between image acquisition time and noise level. Laetitia et al. (18) showed that IQ-SPECT increases the photon sensitivity and contrast-noise ratio more than conventional SPECT. Ali et al. (19) demonstrated that the application of reconstruction algorithm with resolution recovery reduces the acquisition time, although the diagnostic accuracy remains the same. Our results also showed that the use of IQ-SPECT reduces the acquisition time, required for ^201^Tl studies.

The long effective half-life of ^201^Tl limits the dose that can be injected. Therefore, ^201^Tl studies usually require a long acquisition time in order to secure sufficient image quality in comparison with ^99m^Tc studies. Furthermore, we performed conventional SPECT using a triple-head gamma camera. Although these cameras exhibit higher sensitivity compared to dual-head cameras, the IQ-SPECT protocol in MPI required a shorter acquisition time compared to conventional SPECT, using a triple-head camera. Our results suggest that the application of IQ-SPECT also confers advantages with regard to patient comfort, and potentially reduces patient motion. Moreover, this technique may also offer the opportunity to reduce the injected dose to the patient.

There are several limitations inherent in this study. Firstly, in order to avoid introducing additional variation from system-specific correction methods, neither scatter nor attenuation correction was performed. Compton scattering and photon absorption are particularly important factors since organs of significantly different densities (20) surround the heart.

Viji et al. (21) reported that energy-window-based scatter estimate and CT-based attenuation correction are included in IQ-SPECT. Therefore, the use of scatter and attenuation correction may result in different performance outcomes with respect to the level of quantitative segmental tracer uptake. Therefore, further studies are required to examine image quality with attenuation and scatter correction.

Secondly, this study only examined the efficacy of rest MPI SPECT. Patient and/or organ motion such as upward creeping of the heart is often observed after exercise stress (22), and such motions during image acquisition can lead to serious reconstruction artifacts. Finally, no assessment of diagnostic accuracy was performed, although evaluation of diagnostic accuracy is necessary for determining the clinical usefulness of IQ-SPECT.

## Conclusion

In this study, IQ-SPECT with shorter acquisition time provided MPI SPECT images, comparable to those of conventional SPECT, using a triple-head gamma camera, according to ^99m^Tc and ^201^Tl protocols. Our results suggest that IQ-SPECT is a beneficial technology for performing MPI SPECT, and this modality is expected to increase scan efficiency and improve patient comfort.
